# Brevenal, a Marine Natural Product, is Anti-Inflammatory and an Immunomodulator of Macrophage and Lung Epithelial Cells

**DOI:** 10.3390/md17030184

**Published:** 2019-03-20

**Authors:** Devon M. Keeler, Meghan K. Grandal, Jennifer R. McCall

**Affiliations:** UNCW Center for Marine Science, 5600 Marvin K Moss Lane, Wilmington, NC 28409, USA; dmk9506@uncw.edu (D.M.K.); grandal@musc.edu (M.K.G.)

**Keywords:** brevenal, marine natural product, immunomodulator, anti-inflammatory, cystic fibrosis, COPD, asthma, cytokine, chemokine, macrophage activation, macrophage phenotype, mucociliary clearance

## Abstract

Chronic respiratory diseases, including chronic obstructive pulmonary disease (COPD), cystic fibrosis, and asthma, are some of the leading causes of illness and fatalities worldwide. The search for novel treatments led to the exploration of marine natural products as drug candidates to combat the debilitating effects of mucus accumulation and chronic inflammation. Previous research showed that an alga-derived compound, brevenal, could attenuate the effects of inflammatory agents, but the mechanisms by which it exerted its effects remained unclear. We investigated the effects of brevenal on lipopolysaccharide (LPS) induced cytokine/chemokine production from murine macrophages and human lung epithelial cells. It was found that brevenal reduces proinflammatory mediator secretion while preserving anti-inflammatory secretion from these cells. Furthermore, we found that brevenal does not alter cell surface Toll-like receptor 4 (TLR4) expression, thereby maintaining the cells’ ability to respond to bacterial infection. However, brevenal does alter macrophage activation states, as demonstrated by reduced expression of both M1 and M2 phenotype markers, indicating this putative anti-inflammatory drug shifts innate immune cells to a less active state. Such a mechanism of action would be ideal for reducing inflammation in the lung, especially with patients suffering from chronic respiratory diseases, where inflammation can be lethal.

## 1. Introduction

Chronic respiratory diseases are some of the leading causes of illness and death globally [1]. Chronic obstructive pulmonary disease (COPD), cystic fibrosis (CF), and asthma can all be fatal diseases that affect lung function by increasing inflammation and decreasing mucus clearance. Inflammation is a natural biological response, and at a local site of damage or infection, it is an important mechanism for the immune system to clear harmful stimuli. However, excessive inflammation, like that which occurs during sepsis, can damage delicate organ structures, such as those found in the respiratory system. Prolonged inflammation and corresponding damage of critical organ systems can be lethal [2,3]).

Millions of people suffer globally from chronic respiratory diseases, including estimates of 65 million with COPD, 300 million with asthma, and 70,000 with CF [1,4,5,6,7,8,9]. Approximately 3 million people die each year from COPD [1,4,5]. Regrettably, it is estimated that 250,000 deaths are attributed to asthma each year, many of which could have been prevented with sufficient medical intervention [9]. Life expectancy of a patient with CF was only 5 years in the 1960s, and while the quality and length of life have improved dramatically since then, CF remains a debilitating and ultimately fatal disease [6,7,8]. As these diseases continue to have such an aggressive impact on human mortality, it is critical to progress research into the causes, prevention, and new treatments of these diseases.

**Figure 1:**
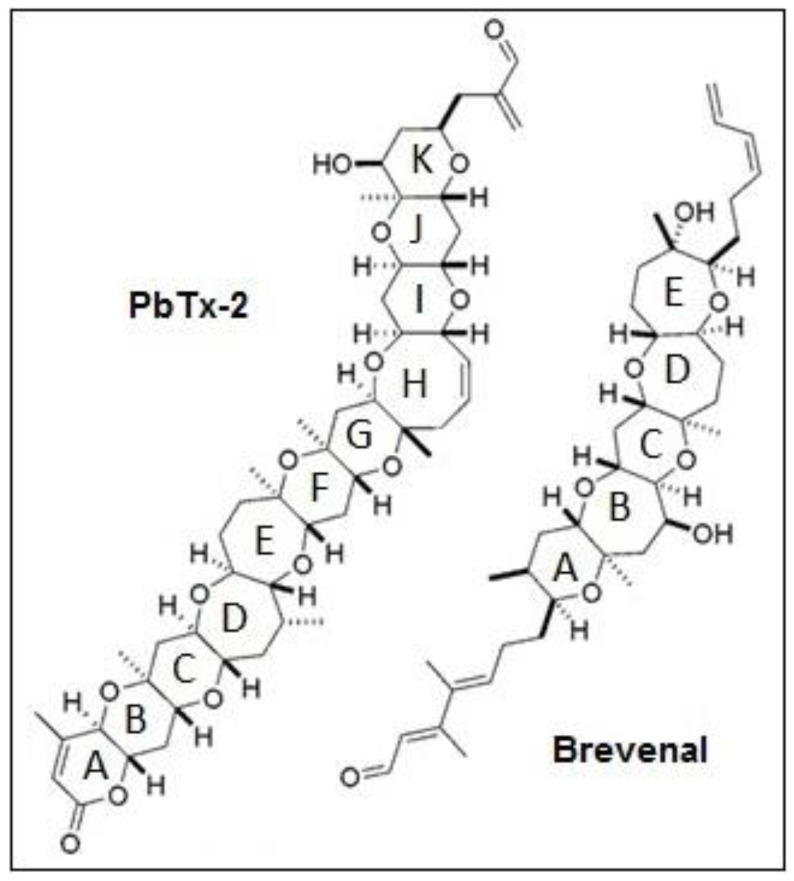
Chemical structures of relevant *Karenia brevis* natural products.

Searching for novel treatments for these diseases requires exploring new drug candidates with bioactivity that can combat inflammation and mucus accumulation. The marine environment provides a diverse source of natural products with potential to treat human ailments. The marine dinoflagellate *Karenia brevis* is one such source of bioactive compounds. This unicellular alga produces a number of bioactive compounds with therapeutic potential, including the neurotoxic brevetoxins (PbTxs), hemibrevetoxin B, brevisin, brevisamide, tamulamides A and B, and brevenal [10,11,12,13,14,15,16]. Brevenal (Figure 1) was the first natural nontoxic ligand described that displaces PbTxs from binding to voltage-sensitive sodium channels [16]. In cell models, brevenal has been found to antagonize PbTx-induced elevations in intracellular calcium levels [17] and reduce cell death in the presence of highly toxic concentrations of PbTxs [18]. In vivo models have shown that brevenal can attenuate PbTx-induced bronchoconstriction and increase tracheal mucosal velocity in sheep [19]. The ability of brevenal to increase tracheal mucosal velocity and mucocilliary clearance has led to the patenting of brevenal as a treatment for COPD, cystic fibrosis, and asthma, as well as attempts by Silurian Pharmaceuticals to begin Phase I clinical testing for the treatment of cystic fibrosis.

During the in vivo investigation of the antagonistic effects of brevenal against brevetoxins, it was discovered that brevenal alone could also attenuate the effects of other inflammatory agents. For example, brevenal has been shown to attenuate neutrophil elastase-induced bronchoconstriction and decrease neutrophil recruitment into the lung [20,21,22]. These results indicate anti-inflammatory effects not typically seen with traditional lung clearing pharmaceuticals [20,21,22,23]. Brevenal has also been found to attenuate PbTx-induced activity and sodium influx in, but not activation of, mast cells, a key immune cell that coordinates allergic responses [24]. While brevenal shows promise as a potential therapeutic for lung disease, the mechanism by which brevenal can attenuate inflammation remains unclear.

Secondary to airway restriction, chronic respiratory diseases are often compounded by the effects of inflammation. An excessive inflammatory response can cause serious damage to lung tissues, decreasing quality of life and increasing debilitating symptoms associated with COPD, asthma, and CF. As such, ideal drug candidates for chronic respiratory disease will have a dual effect: Combat the root cause of disease (e.g., bronchoconstriction or mucus accumulation) and simultaneously reduce damaging inflammation. The purpose of our study was to examine the effects of brevenal on pro- and anti-inflammatory cytokine production from lung epithelial cells and immune cells, as an additive mechanism to its influence on airway restriction. Macrophages were used for this study because of their role in coordinating inflammatory responses, both in the lung and systemically. We further examined the effects of brevenal on phenotypic markers of macrophage activation to determine the mechanisms by which brevenal exerts an anti-inflammatory response, thereby demonstrating its utility for treating chronic respiratory diseases.

## 2. Results

### 2.1. Brevenal is not Toxic for A549 Epithelial Lung Cells, MH-S Lung Macrophages, or RAW 264.7 Macrophages at Micromolar Concentrations

**Figure 2:**
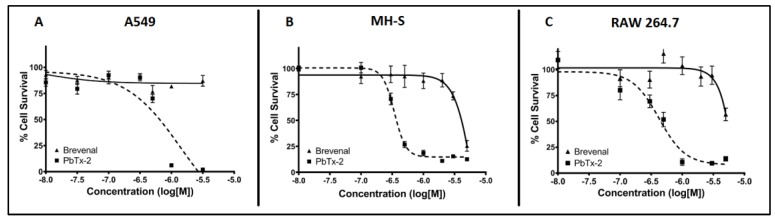
Brevenal does not induce cytotoxicity of model cell lines at targeted treatment concentrations. Model cell lines were assessed for percentage cell death using fluorescence microscopy. Panel **A** shows the A549 lung epithelial cell line, panel **B** shows the MH-S lung macrophage cell line, and panel **C** shows the RAW 264.7 macrophage cell line. Brevenal did not demonstrate toxicity in any model cell line at or below 100 nM (−7 in log[M]).

Cytotoxicity assays were performed to assess the potential toxicity of brevenal on model cell lines to ensure toxicity would not be an extraneous variable in results. As shown in Figure 2, brevenal did not induce cell death in A549 epithelial lung cells (Figure 2A), MH-S lung macrophages (Figure 2B), or RAW 264.7 macrophages (Figure 2C) up to 100 nM. All further studies were completed in these cell lines with concentrations of brevenal of 100 nM (−7 in log[M]) or less.

### 2.2. Brevenal Decreased LPS-Induced Production of the Proinflammatory Cytokine IL-8 from Human Lung Cells

**Figure 3:**
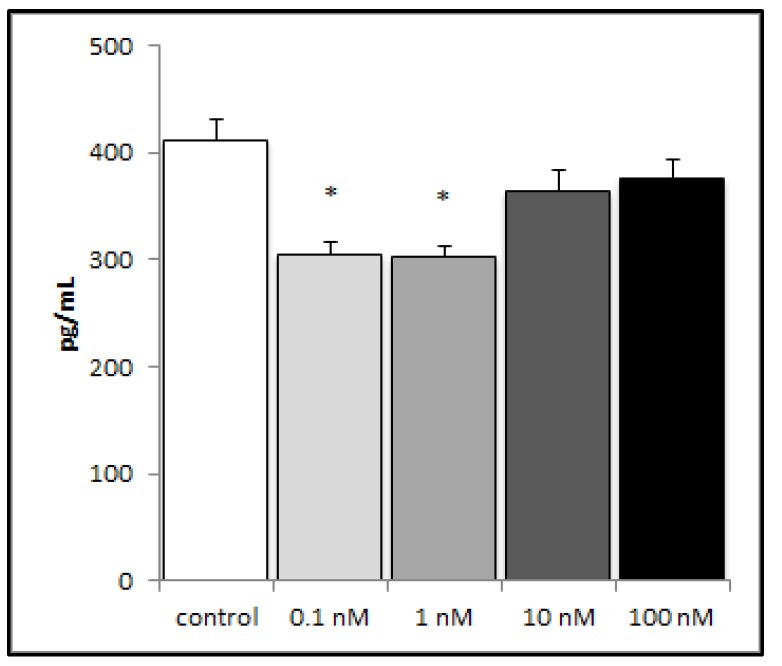
LPS-induced production of IL-8 from A549 cells decreased with brevenal treatment. LPS-induced production of IL-8 significantly decreased with 24 h brevenal pretreatment (0.1 and 1 nM) as compared to the ethanol control. Results are presented as mean +/− standard error, and * indicates statistical significance from control of p < 0.05 (n = 8).

In order to investigate the potential influence of brevenal on the inflammatory response in lung tissue, we challenged the human lung epithelial cell line (A549) with lipopolysaccharide (LPS) (an inflammatory stimulus), and then measured inflammatory mediator production. A549 cells did not produce detectable levels of TNFα (data not shown), either with or without brevenal. A549 cells did produce the proinflammatory chemokine IL-8, which decreased significantly with pretreatment of nanomolar concentrations of brevenal (Figure 3).

### 2.3. Brevenal Decreased LPS-Induced Production of Proinflammatory Cytokines from Murine Macrophages but Did Not Alter Pleiotropic or Anti-Inflammatory Cytokine Production

**Figure 4:**
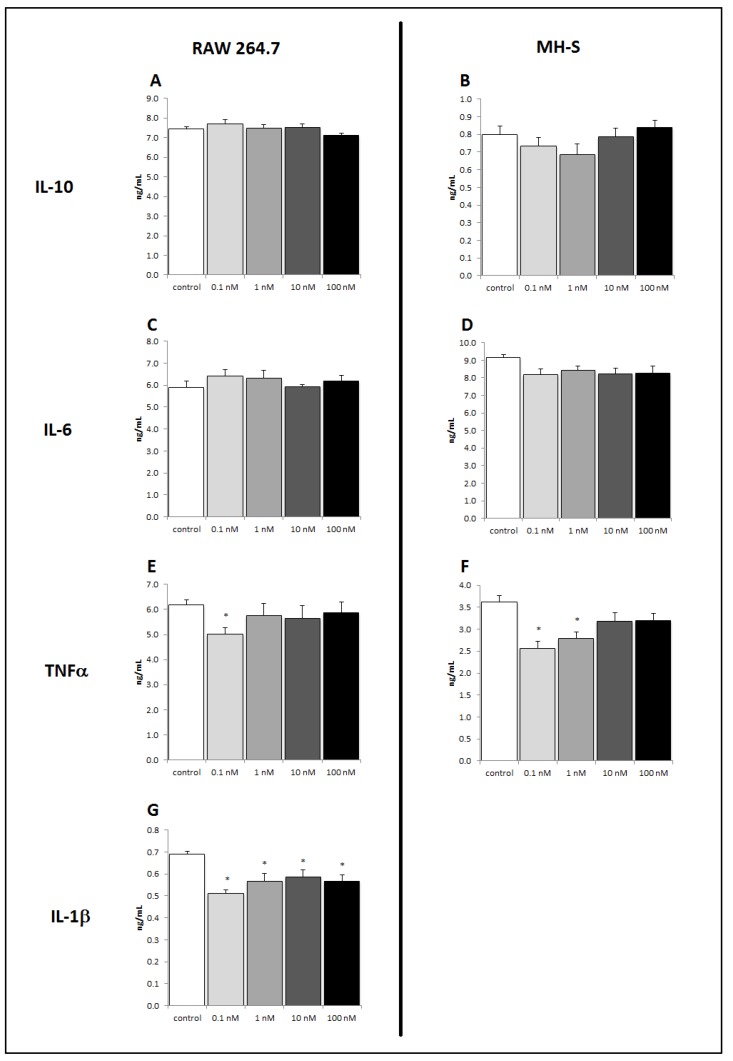
Brevenal decreased LPS-induced production of proinflammatory TNFα and IL-1β without altering IL-6 or IL-10 secretion from macrophage cell lines. Macrophage cell lines were assessed for pro- and anti-inflammatory cytokine secretion following LPS stimulation and pretreatment of brevenal. Secretion from RAW 264.7 macrophages is shown for IL-10 (4**A**), IL-6 (4**C**), TNFα (4**E**), and IL-1β (4**G**). Secretion from MH-S macrophages is shown for IL-10 (4**B**), IL-6 (4**D**), and TNFα (4**F**). Data are shown as mean +/− standard error, and * indicates statistical significance from control of p < 0.05 (n = 8).

Resident macrophages in the lung can coordinate inflammatory responses to infection or disease. To further examine the effects of brevenal on immune responses, we investigated this putative anti-inflammatory therapeutic on two monocyte/macrophage cell lines. MH-S lung macrophages and RAW 264.7 macrophages were stimulated with LPS following brevenal pretreatment. The potent proinflammatory cytokines IL-1β and TNFα were measured, as was the pleiotropic cytokine IL-6 and the anti-inflammatory cytokine IL-10. MH-S cells demonstrated a significant decrease in LPS-induced TNFα production at 24 h of 0.1 nM brevenal treatment (Figure 4F). However, there was no significant change in LPS-induced IL-6 or IL-10 production with brevenal pretreatment (Figure 4B,D). RAW 264.7 cells demonstrated a significant decrease in LPS-induced IL-1β and TNFα production following brevenal treatment (Figure 4E,G). Similar to MH-S cells, RAW 264.7 cells did not show a significant change in LPS-induced IL-6 or IL-10 secretion (Figure 4A,C). It is important to note that brevenal treatment alone, in absence of LPS stimulation, did not elicit any measurable cytokine secretion from macrophage cell lines (data not shown).

**Figure 5:**
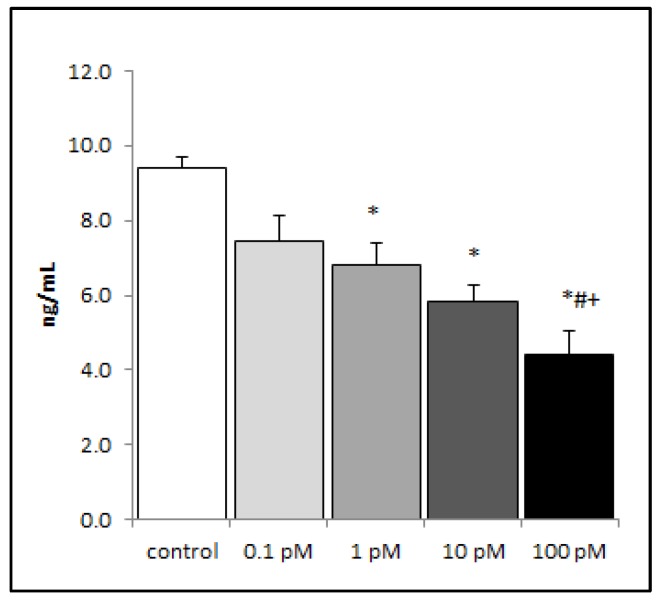
Brevenal decreased LPS-induced production of proinflammatory TNFα secretion at very low doses. Macrophage cell lines were assessed for TNFα cytokine secretion following LPS stimulation and pretreatment of brevenal. Data are shown as mean +/− standard error, and * indicates statistical significance from control, # from 0.1 pM, and + from 1 pM, all of p < 0.05 (n = 7–8).

To discover a lower limit of effect, RAW 264.7 macrophages were treated with 1000-fold lower doses of brevenal and then stimulated with LPS. As shown in Figure 5, brevenal significantly inhibited LPS-induced TNFα secretion down to 1 pM concentration. Brevenal was unable to attenuate LPS-induced IL-1β secretion at the same very low concentrations (data not shown).

### 2.4. Treatment of RAW 264.7 Cells with Brevenal Did Not Change Cell-Surface Expression of TLR4, with or without LPS Co-Stimulation

**Figure 6:**
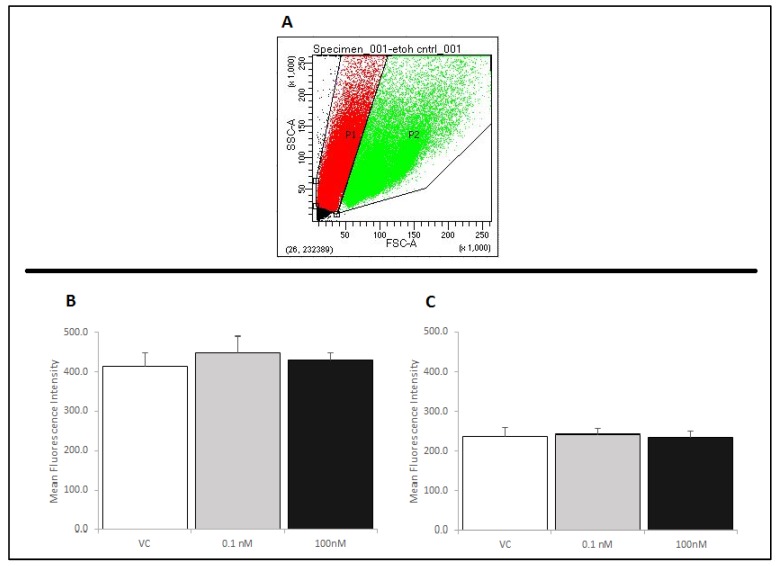
RAW 264.7 cells were assessed for changes in TLR4 expression by flow cytometry. Cells were assessed for size and gated for a single cell population (P1) using a forward and side scatter dot plot (Panel **A**). Panels **B** and **C** show relative fluorescence intensity compared across treatments as a measure of TLR4 receptors at the membrane. Neither brevenal alone (6**B**) nor brevenal combined with LPS (6**C**) caused a change in TLR4 expression when compared to ethanol controls (VC). Data are shown as mean +/− standard deviation (n = 4).

In order to determine a potential mechanism by which brevenal exerts its anti-inflammatory actions, we assessed RAW 264.7 cells for Toll-like receptor 4 (TLR4) expression by flow cytometry. Figure 6A shows the size and complexity of cells without brevenal or LPS treatment, as assessed by a forward and side scatter dot-plot. The RAW 264.7 cells used in these experiments exhibited two distinct populations (P1 and P2). Cells were gated and each population was analyzed separately, as RAW 264.7 cells have been shown to exhibit multiple populations if samples are not filtered prior to analysis. The larger sized signals (P2) are likely caused by cell aggregation [25,26]. To avoid effects due to aggregation, the single cell population (P1) was gated from the aggregate cell population (P2), and both populations were separately assessed for TLR4 expression using a PE-conjugated anti-TLR4 antibody. It was determined that brevenal caused no significant changes in the cell-surface expression of TLR4 receptors, with or without LPS co-stimulation in single cells (Figure 6B,C). Similar to P1, the P2 population of cells did not show significant changes in the cell-surface expression of TLR4 receptors (data not shown).

### 2.5. Brevenal Treatment Decreased Expression of Markers for Both M1 and M2 Phenotypes on RAW 264.7 Cells, Decreased Cell Size, and Decreased Cell Complexity

**Figure 7:**
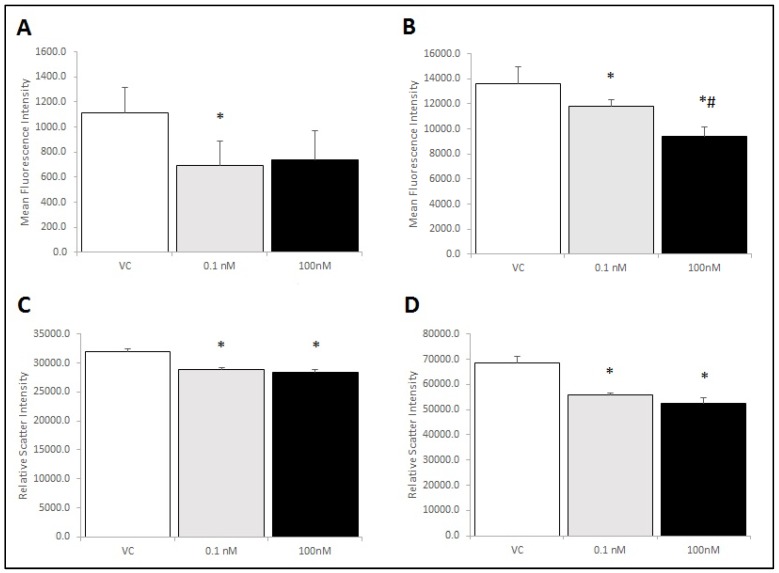
RAW 264.7 cells were assessed for activation phenotype following brevenal treatment. Cells were co-stained for activation markers (M1: CD86 and M2: CD206) and assessed for changes in size (forward scatter) and complexity (side scatter) in response to brevenal treatment. Cells were gated and analyzed to determine effects on the single cell population (P1). Panel **A**: Average fluorescence intensity for CD86. Panel **B**: Average fluorescence intensity for CD206. Panel **C**: Forward scatter as a measurement of cell size. Panel **D**: Side scatter as a measurement of cell complexity. Data are shown as mean +/− standard deviation, while * indicates statistical significance from control (VC) and # indicates statistical significance from 0.1 nM of p < 0.05 (n = 4).

To examine potential mechanisms by which brevenal could elicit changes in macrophage activation states, RAW 264.7 cells were treated with brevenal and assessed for cell-surface markers of activation. The classical activation/pro-inflammatory cytokine secretion phenotype (M1) was assessed using the cell-surface marker CD86. The alternative activation phenotype (M2) was assessed using the mannose receptor (CD206). As seen in Figure 7A,B respectively, CD86 and CD206 markers decreased significantly with brevenal treatment in the single cell population (P1). Brevenal treatment did not alter activation phenotype markers in the aggregate cell population (P2). Forward scatter and side scatter were also significantly decreased with brevenal treatment (Figure 7C,D respectively), indicating a decrease in cell size and complexity in the single cell population (P1). Brevenal treatment did not alter forward scatter or side scatter in (P2), the aggregate cell population (data not shown).

### 2.6. Brevenal Treatment Alters the Percentage of RAW 264.7 Cells that are Aggregated in Culture

Brevenal was shown to have effects in the single cell gated population (P1) of RAW 264.7 cells, but not the aggregate cell gated population (P2). Brevenal reduced cell size and complexity, as well as activation phenotypes, in single cells. Cells have been known to aggregate in response to inflammatory stimuli because of increased expression of adhesion molecules. To determine if brevenal affected the rate of P1 and P2, we examined the percentage of cells in each population as compared to the total counts calculated in flow cytometry experiments (see Figure 6A for the gating scheme). As shown in Table 1, a low dose of brevenal significantly reduced the percentage of cells in the larger aggregate cell group. There was a corresponding increase in the percentage of cells in the smaller single cell group, but this change did not reach the level of statistical significance.

## 3. Discussion

Chronic respiratory diseases are a leading cause of death globally, and include COPD, CF, and asthma [1]. These diseases are exacerbated by inflammation, which can inhibit lung function and cause lung damage. Ideal novel treatments for chronic respiratory diseases dually address mucus clearance and inflammation. Brevenal, a natural product isolated from marine algae, has been shown to increase mucociliary clearance in animal models of chronic respiratory disease [19]. Brevenal also demonstrated the potential for anti-inflammatory action not typically seen with traditional lung clearing pharmaceuticals [20,21,22,23]. However, these previous studies were unable to determine the mechanism of action by which brevenal reduced lung inflammation.

The purpose of our study was to examine the anti-inflammatory effects of brevenal in the lung, and to determine potential mechanisms of action of this putative respiratory therapeutic. Native lung epithelial cells and cell lines (such as A549 cells) are known to produce cytokines and chemokines in response to inflammatory stimuli that recruit inflammatory cells to the site of infection or damage. IL-8 is a proinflammatory chemokine that attracts neutrophils to sites where an immune response has been initiated, where they then propagate the inflammatory reaction [27]. Our results indicate that brevenal can reduce inflammatory cytokine secretion from lung epithelial cells after exposure to an inflammatory stimulus, thereby potentially reducing an exacerbation of inflammation. These results are similar to what has been found with corticosteroid treatment, where budesonide reduced IL-8 secretion from bronchial epithelial cells [28].

Additionally, our studies examined brevenal’s anti-inflammatory action in macrophages. Macrophages respond to harmful stimuli and cytokines to propagate immune responses by amplifying chemical signals, making them pivotal in the initial immune response [29]. TNFα is a proinflammatory cytokine that initiates the innate immune response by influencing other cells to produce more proinflammatory cytokines. Inflammatory diseases (e.g., sepsis and rheumatoid arthritis) are often characterized by excessive production of TNFα [30]. In addition to TNFα, we measured macrophage production of IL-1β, a potent proinflammatory cytokine associated with immunopathologies [31], IL-6, a pleiotropic cytokine with pro- and anti-inflammatory influences [32], and IL-10, an anti-inflammatory cytokine that suppresses the immune response and prevents tissue damage [33]. Our data indicate that brevenal acts as an anti-inflammatory agent due to its ability to reduce proinflammatory cytokine secretion and not affect production of pleiotropic or anti-inflammatory cytokines. One would not expect nor desire a potential lung therapeutic to have a severe effect on the ability of immune sentinel cells to respond to stimuli and produce cytokines. We suggest that brevenal would have a modest but significant physiological effect in only partially reducing inflammatory responses. Key to this suggestion is that brevenal affects the most potent inflammatory cytokines (i.e., IL-1β and TNFα), which are particularly problematic in inflammatory diseases [32]. However, brevenal does not unilaterally inhibit secretion of all cytokines. IL-6 is considered pleiotropic and immunomodulatory because it can have multiple effects, including stimulating the immune response to trauma and activating lymphocytes [32]. Indeed, other anti-inflammatory drugs, such as corticosteroids, have been found to inhibit an entire suite of cytokines, from the initial phase inflammatory mediators IL-1 β and TNFα, to immunomodulatory interleukins (including IL-6), to even the anti-inflammatory IL-10 [34]. Our study found that brevenal reduced inflammatory cytokine secretion by 20%–35%. The corticosteroid budesonide has been found to reduce TNFα secretion from macrophages in vitro by approximately 40% at a similar dose of 1 nM [35]. Additionally, it has been found that serum levels of IL-1β and TNFα are only 45%–50% lower in healthy patients than those hospitalized with sepsis [36]. As such, we suggest that our results would translate in vivo to a measured yet significant physiological effect to reduce damaging inflammation in lung disease, without compromising the necessary healing response of the immune system. However, further studies would need to be completed to fully demonstrate such an effect.

Our results also indicate a biphasic response to brevenal in cytokine secretion, where lower doses are more effective than higher doses. While the mechanisms behind this effect are not completely clear at this time, such biphasic responses are not uncommon in pharmaceutical agents. Indeed, marine-derived compounds have previously been shown to have biphasic responses, including other compounds from the same algal source as brevenal [37,38]. Further studies would be needed to determine the exact reason for such biphasic effects, though it could be due to a development of tolerance by the cells at higher doses of brevenal. Regardless, brevenal is primarily advocated as therapeutic for lung diseases in which mucocilliary clearance is the target. Any anti-inflammatory effects would be secondary and synergistic to the primary effect of increasing tracheal mucosal velocity and mucocilliary clearance. Indeed, it has been suggested that general anti-inflammatory effects are a leading reason why brevenal is believed to be superior over current CF treatments [22]. As a treatment for lung clearance, however, brevenal has been stated to be ineffective at doses below 0.1 nM [39], making doses below this less physiologically relevant for either use.

Brevenal’s ability to reduce proinflammatory cytokine secretion led us to investigate its effect on macrophage cell surface receptor expression as a possible mechanism for these observations. First, we examined TLR4 expression because it binds LPS and initiates the innate immune response [40]. However, our results do not indicate that brevenal alters TLR4 expression or the ability of macrophages to respond to bacterial infection via the expression of this receptor. As brevenal therefore must act through another mechanism, we examined the ability of brevenal to alter activation states of macrophages. The M1 phenotype is the classical activation pathway associated with response to infection and production of proinflammatory cytokines [41,42,43]. Here, we show that brevenal treatment reduced the M1 phenotype marker (CD86) in macrophages, demonstrating a mechanism for suppression of inflammatory activation. The M2 phenotype is indicative of the alternative activation pathway associated with wound and tissue healing [41,42,43]. Brevenal reduced the M2 phenotype marker (CD206) and also reduced cell size and complexity of macrophages.

Interestingly, our results only extend to the single cell population of RAW 264.7 cells, as brevenal did not have an effect on activation states or size and complexity in aggregated cells. The mechanisms behind this finding are not immediately clear, but aggregation alone could certainly have confounding effects. It could be that brevenal cannot overrule an already activated macrophage, and therefore has greater utility as a prophylactic than as a rescue therapeutic for lung inflammation. If patients are already using brevenal to increase mucocilliary clearance, then it could have an added synergistic effect of a modest reduction in pulmonary inflammation. Such an effect would be ideal to maintain the ability of cells to respond to infection. Additionally, we found that brevenal reduced the percentage of cells in the aggregated population at low doses. As such, brevenal could have an effect on the ability of cells to aggregate, but further studies would be needed to confirm this hypothesis.

Overall, our results suggest that brevenal treatment elicits an anti-inflammatory response in epithelial cells and macrophages in addition to improving mucociliary clearance shown in previous studies. The mechanism of its immunomodulatory effect is through decreased activation of macrophages in general, putting them in a so-called quiet state, rather than shifting phenotype. This mechanism of action is ideal for reducing lung inflammation, especially in patients with chronic respiratory diseases where inflammation can be severely damaging and potentially lethal. These findings support further investigation on the utility of brevenal as a dual-action therapeutic for life threatening pulmonary diseases, including COPD, asthma, and CF.

## 4. Materials and Methods

### 4.1. Cell Culture

Human alveolar epithelial cells (A549, CCL-185), murine alveolar macrophage cells (MH-S, CRL-2019), and murine macrophage-like cells (RAW 264.7, CRL-2278) were utilized in this study (ATCC, Manassas, VA, USA). A549 cells were cultured in F12K medium, MH-S in RPMI 1640 medium, and RAW 264.7 in DMEM with Glutamax (Thermo Fisher Scientific, Waltham, MA, USA). All media contained 10% FBS and penicillin/streptomycin (Thermo Fisher Scientific, Waltham, MA, USA).

### 4.2. Brevenal Treatment

Cells were treated with brevenal acquired from Dr. Daniel Baden, extracted from *K. brevis* cultures as previously described [16]. A certificate of purity was obtained, demonstrating purity of greater than 95%, as determined by high pressure liquid chromatography analysis (single peak elution from C-18 reverse phase column, UV detection at 215 nm and 290 nm, mobile phase 89% methanol and 11% water). Doses of treatment ranged from 0.1 pM to 100 nM. The choice of doses was based on toxicity studies and previous studies and efficacy of brevenal as a treatment for increasing tracheal mucosal velocity and mucocilliary clearance, as any anti-inflammatory effect would be additive to its use as a CF therapy. Previous studies found efficacy of brevenal from 1 ng/mL (1.52 nM equivalent) to 50 µg/mL (76.22 µM equivalent) [20,21,22,39]. Additionally, brevenal has been found to be ineffective below 1 ng/mL [39]. Previous studies with corticosteroids have used similar ranges of 0.1 nM to 100 nM [28].

### 4.3. Cytotoxicity Assays

Cytotoxicity assays were conducted for each cell line as previously described [38]. Briefly, cells were seeded into 96-well plates, incubated at 37 °C for 24 h, and treated with brevenal, positive control (PbTx-2), or negative control of absolute ethanol (Fisher Scientific, Waltham, MA, USA). Brevenal and PbTx-2 were extracted from *K. brevis* cultures grown by colleagues at UNCW as described previously [16]. Cells were treated with a nuclear stain, Hoechst 33342 (Invitrogen Life Technologies, Waltham, MA, USA) for 24 h. Fluorescence was measured using the DAPI filter on an Image Xpress Micro (Molecular Devices, LLC; San Jose, CA, USA). Four images per well were acquired and analyzed with MetaXpress Image Acquisition and Analysis v2.0.1.44 software (Molecular Devices, LLC; San Jose, CA, USA).

### 4.4. Quantification of Pro- and Anti-Inflammatory Cytokines

A549, MH-S, and RAW 264.7 cells were seeded in 6-well plates, treated with brevenal [0-100 nM], and incubated at 37 °C for 24 h before stimulation with 400 ng/mL lipopolysaccharide (LPS) (Sigma-Aldrich; St. Louis, MO, USA). Supernatant was collected and stored at −20 °C until enzyme linked immunosorbent assays (ELISAs) were performed to measure cytokine production, using methods described previously [44]. For murine lines (RAW 264.7 and MH-S), IL-1β, IL-10, and TNFα DuoSet^®^ ELISA kits (R&D Systems, Inc.; Minneapolis, MN, USA) were used to detect cytokines production with brevenal and LPS treatment. Antibodies were used for the detection of murine IL-6 (BD Pharmingen^TM^, San Diego, CA, USA). For human A549 cells, human IL-8 and TNFα, DuoSet^®^ ELISA kits (R&D Systems, Inc.; Minneapolis, MN, USA) were used. Absorbance was read at 450 nm using a FlexStation III plate reader (Molecular Devices, LLC; San Jose, CA, USA).

### 4.5. Flow Cytometry Assessment for Toll-Like Receptor 4 (TLR4), Mannose Receptor (CD206), and CD86 Receptor Expression

RAW 264.7 macrophages were seeded in 6-well plates and incubated at 37 °C until cells adhered and grew to confluence before being treated with ethanol (negative control), brevenal, or brevenal and LPS. Antibody staining and flow cytometry experiments were initiated following a 24 h incubation with treatment at 37 °C. Cells were harvested, centrifuged, and media were removed. The cells were stained on ice for 1 hr with 2 μg/mL (TLR4) or 4 μg/mL (CD206 or CD86) fluorescent antibody solution. Unbound antibody was removed and cells were resuspended in cold PBS prior to analysis on a BD FACSCelesta^TM^ flow cytometer (BD Biosciences; San Jose, CA, USA).

Expression of Toll-like receptor 4 (TLR4 or CD284/MD-2) was measured using phycoerythrin (PE)-conjugated rat anti-mouse antibodies directed against CD284/MD-2 (MTS510; BD Pharmingen^™^, San Diego, CA, USA). Expression of mannose receptors (CD206) was measured using Alexa Fluor^®^ 488-conjugated rabbit anti-mouse antibodies directed against CD206 (clone EPR6828(B); abcam, Cambridge, MA, USA). Expression of CD86 receptors was measured using allophycocyanin (APC)-conjugated rat anti-mouse antibodies directed against CD86 (clone GL-1; abcam, Cambridge, MA, USA).

### 4.6. Statistical Analyses

Cell viability was determined by image analysis of Hoechst 33342 nuclear dye. Cell counts were averaged from four images per well. Cytotoxicity was calculated as the percentage of viable cells in treatment wells compared to control wells. Cytotoxicity calculations from 96-well images were automated by SAS v9.1.3 software (SAS Institute Inc., Cary, NC, USA) and non-linear regression curve-fit was performed in GraphPad Prism v4.03 (GraphPad Software, San Diego, CA, USA). Data points are averages of three replicate 96-well plate experiments.

Cytokine concentration was measured by absorbance values fit to a standard curve. IL-8 was quantified in pg/mL and IL-1β, TNFα, IL-6, and IL-10 were quantified in ng/mL. Values for each treatment were averaged and standard deviation and standard error were calculated. One-way ANOVAs were performed using JMP Pro v14.0 with post hoc analysis to determine statistical significance (p < 0.05) (SAS Institute Inc., Cary, NC, USA).

For flow cytometry experiments, the mean values for fluorescence intensity and/or forward scatter and side scatter were recorded for each sample and averages were calculated within treatments. Standard deviation and standard error were calculated, and one-way ANOVAs were performed using JMP Pro v14.0 with post hoc analysis, including Tukey’s all pairs tests, to determine statistical significance (p < 0.05) (SAS Institute Inc., Cary, NC, USA).

## Figures and Tables

**Table 1:** Brevenal reduces the percentage of cells in P2. Data are presented as the mean +/− standard deviation of the percentage of cells in gated populations of total counts.

Treatment	% P1	% P2
VC	55.2% +/− 1.9%	40.0% +/− 2.4%
0.1 nM	57.9% +/− 3.5%	34.1% +/− 3.4% (*)
100 nM	55.9% +/− 1.7%	34.9% +/− 2.7%

* indicates statistical significance from vehicle control (n = 4).
